# Heat-Treated *Limosilactobacillus fermentum* PS150 Improves Sleep Quality with Severity-Dependent Benefits: A Randomized, Placebo-Controlled Trial

**DOI:** 10.3390/nu18010014

**Published:** 2025-12-19

**Authors:** Mon-Chien Lee, Chao-Yuan Chen, Ching-Yun Chen, Chi-Chang Huang

**Affiliations:** 1 Graduate Institute of Sports Science, National Taiwan Sport University, Taoyuan City 333325, Taiwan; 2 Center for General Education, Taipei Medical University, Taipei 110301, Taiwan; 3 Physical Education Office, National Taipei University of Business, Taipei 100025, Taiwan

**Keywords:** sleep quality, postbiotic, *Limosilactobacillus fermentum* PS150, gut–brain axis, wrist actigraphy

## Abstract

**Background**: Insomnia is prevalent and difficult to treat safely over the long term. Given the role of the microbiota–gut–brain axis in melatonin and hypothalamic–pituitary–adrenal (HPA) regulation, and preclinical evidence for *Limosilactobacillus fermentum* PS150, we evaluated whether a heat-treated formulation (HT-PS150) could improve sleep and modulate endocrine/circadian markers in adults with poor sleep. **Methods**: In a randomized, double-blind, placebo-controlled trial, 84 adults aged 20–60 years with PSQI ≥ 5 and ISI < 22 were assigned to receive either placebo or HT-PS150 for eight weeks. Outcomes included patient-reported sleep (PSQI, ISI), anxiety/depression (GAD-7, PHQ-9), quality of life (QLESQ-SF), gastrointestinal symptoms (VAS-GI), wrist actigraphy (Fitbit Inspire 3), and sleep-relevant biomarkers measured from urine, saliva, and/or blood samples (melatonin, cortisol, orexin, serotonin, GABA, and/or norepinephrine). Repeated measures were analyzed using generalized estimating equations. An exploratory proportional regulation analysis classified individual biomarker changes as up- or down-regulated and compared proportions between study arms. Per-protocol analyses required ≥80% compliance. **Results**: Improvements in the primary outcomes, PSQI and ISI, were observed over time in both groups, while no significant group × time interactions were detected. In exploratory proportional analyses, a higher proportion of participants in the HT-PS150 group exhibited up-regulated nocturnal melatonin secretion and improved daytime plasma orexin levels, as well as a tendency toward greater reductions in nocturnal salivary cortisol compared with placebo. In subgroup analyses with higher baseline insomnia severity (ISI ≥ 8), HT-PS150 was associated with greater improvements in PSQI (notably sleep duration and efficiency) and reduction in anxiety (GAD-7) upon post hoc testing. **Conclusions**: Although group mean scores on sleep symptom scales did not differ significantly in the full cohort, HT-PS150 appeared to modulate sleep–wake regulation by enhancing nocturnal melatonin secretion, attenuating HPA-axis activity, and stabilizing wakefulness. Clinical benefits were most evident among participants with greater baseline symptom burden, suggesting potential utility in more symptomatic populations.

## 1. Introduction

In today’s rapidly changing and stress-laden societies, declining sleep quality has emerged as a critical global public health concern [1]. As a fundamental biological process, sleep is essential for immune homeostasis, neurocognitive performance, and emotional regulation [2]. However, environmental pressures, lifestyle disruptions, and the pervasive influence of digital technologies have markedly increased the burden of sleep disturbances worldwide [3]. Epidemiological evidence indicates that nearly one-third of adults experience insomnia symptoms, with prevalence rising markedly in older populations [4]. Beyond nocturnal symptoms, insomnia contributes to daytime dysfunction, cognitive impairment, emotional disorders, and increased risk of metabolic and cardiovascular diseases [5]. Although pharmacological treatments such as benzodiazepine receptor agonists and melatonin receptor agonists may provide short-term relief, their long-term use is limited by tolerance, dependence, and adverse effects [6], highlighting the need for safer and more sustainable therapeutic strategies.

Given that restorative sleep is fundamental for maintaining overall physical and mental health, the mechanisms underlying sleep regulation have attracted increasing scientific attention. Evidence indicates that sleep quality is strongly influenced by the hypothalamic–pituitary–adrenal (HPA) axis and neuroimmune processes [7], both of which are closely intertwined with the brain–gut–microbiome axis [8]. This bidirectional communication network integrates the central nervous system with the gut microbiome, influencing host metabolism, immune regulation, and neural signaling [9,10]. Gut microbes can synthesize neurotransmitters such as serotonin, dopamine, and γ-aminobutyric acid (GABA), which modulate mood, cognition, and sleep–wake cycles [11]. Notably, approximately 90% of serotonin is produced in the gut, and dysbiosis has been associated with psychiatric conditions including depression, anxiety [11,12,13], and bipolar disorder, often characterized by reduced microbial diversity and depletion of beneficial taxa such as *Faecalibacterium* and *Bifidobacterium* [14]. Beyond mood regulation, microbial metabolites such as short-chain fatty acids can cross the blood–brain barrier, modulate neuroinflammation, and alter neural activity in ways that shape sleep patterns [15]. Conversely, sleep disruption exacerbates gut microbial imbalance, activates the HPA axis, and promotes systemic inflammation, thereby reinforcing sleep disturbances [16,17]. Therefore, modulation of the gut microbiota through the gut–brain axis represents a promising therapeutic approach for improving sleep.

Probiotics are defined as live microorganisms that, when administered in adequate amounts, confer health benefits to the host [18]. More recently, the term “psychobiotics” was introduced to describe probiotic strains with potential applications in mental health, particularly through modulation of the microbiota–gut–brain axis [19,20]. Accumulating evidence suggests that probiotic supplementation can attenuate stress-induced sleep disturbances, alleviate depressive and anxiety symptoms, and improve sleep quality [21,22,23,24]. Clinical trials and meta-analyses have further demonstrated beneficial effects of probiotics, postbiotics (historically termed “paraprobiotics”), and prebiotics in individuals with insomnia or suboptimal sleep, with reported improvements in subjective sleep indices and associated mood states [25]. These benefits are thought to arise from the restoration of microbial diversity, enhancement of gut barrier integrity, modulation of inflammatory cytokines, and regulation of neuroactive compound synthesis, including serotonin and melatonin [26]. Accumulating clinical evidence supports the beneficial role of probiotics and related interventions in sleep regulation [27,28]. However, not all probiotics exert beneficial effects on insomnia, and functional specificity may exist among different strains; thus, further confirmation is warranted.

In addition to live probiotics, increasing evidence has shown that heat-inactivated probiotic preparations can retain critical cell surface components, such as lipoteichoic acids and peptidoglycans, which continue to interact with host pattern recognition receptors while offering improved stability and safety [29,30]. Comparative studies further suggest that non-viable probiotic products may confer health benefits similar to those of live bacteria [31]. The psychobiotic *Limosilactobacillus fermentum* PS150 (PS150), originally isolated from fermented meat sausage, has previously been demonstrated in animal models to suppress inflammation, attenuate activation of the HPA axis, prolong total sleep duration, reduce sleep latency, and prevent caffeine-induced insomnia, while also facilitating the recovery of rapid eye movement (REM) sleep [32,33]. Given the safety, stability, and regulatory advantages of non-viable preparations, a heat-treated derivative of PS150 (HT-PS150) was developed. Preliminary, unpublished observations from our group indicated that HT-PS150 retains sleep-related bioactivity comparable to its live form. Building upon these findings, the present study aimed to evaluate whether eight weeks of supplementation with heat-treated PS150 (HT-PS150) could improve sleep quality. To this end, sleep outcomes were assessed using wearable devices, subjective questionnaires, and a range of biochemical parameters derived from blood, saliva, and urine samples.

## 2. Materials and Methods

### 2.1. Participants

Eligible participants were adults aged 20–60 years with a Pittsburgh Sleep Quality Index (PSQI) score ≥ 5, an Insomnia Severity Index (ISI) score < 22, and a regular lifestyle without shift work at screening (V0). Exclusion criteria included recent use of antibiotics or probiotic supplements (within one month), current treatment with medications or hormones known to affect sleep, uncontrolled hypertension or diabetes, cancer, psychiatric disorders, or other diagnosed sleep disorders. Individuals who were pregnant or lactating, allergic to probiotic products, dependent on tobacco, alcohol, or caffeine, had engaged in long-haul travel across time zones, or had been involved in another interventional trial within the past three months were also excluded. Participants judged unsuitable by the principal investigator were not enrolled. The study was approved by the Institutional Review Board of Landseed International Hospital, Taoyuan, Taiwan (LSHIRB No. 24-005-A2), and conducted in accordance with the principles of the Declaration of Helsinki. All participants provided written informed consent prior to study initiation. The trial was prospectively registered at ClinicalTrials.gov (NCT06486064, 1 August 2024).

### 2.2. Study Procedure

A total of 84 eligible participants were enrolled and randomized into two groups: the placebo group (*n* = 41; one capsule per day containing microcrystalline cellulose) and the HT-PS150 group [ *n* = 43; one capsule per day containing 5 × 10^9^ cells of HT-PS150 powder (Bened Biomedical Co., Ltd., Taipei, Taiwan)]. All participants were instructed to take the assigned capsule once daily after dinner for eight weeks. The randomization list was created using computer-generated numbers using a 1:1 assignment ratio. To maintain equal group sizes, block randomization with a constant block size of four was applied. The allocation process was concealed through sequentially numbered, opaque, and sealed envelopes prepared by an independent staff member who had no involvement in participant recruitment or evaluation. A parallel, between-groups design was chosen rather than a crossover design to minimize potential carryover effects, as gut microbiota and related endocrine responses may persist beyond feasible washout periods. Before (V1) and after (V2) the intervention, participants were required to provide saliva and urine samples, undergo blood collection, and complete questionnaire assessments. During the 8-week supplementation period, in addition to daily capsule intake, participants were also instructed to wear a wrist-worn sleep tracker each night and complete a daily sleep diary. In the per-protocol analysis, individuals with a compliance rate below 80% were excluded. The compliance rate was determined based on capsule counts of unused capsules in the bottles returned by participants at study completion.

### 2.3. Subjective Psychological Inventories

The primary outcomes were changes in sleep quality and insomnia symptoms evaluated by the Pittsburgh Sleep Quality Index (PSQI, [34,35]) and the Insomnia Severity Index (ISI, [36,37,38]). The PSQI is a widely used inventory that evaluates subjective sleep quality during the previous month. A global score (0–21) of 5 or more reflects poor sleep quality. The ISI is a 7-item questionnaire with a total score ranging from 0 to 28. Scores of 0–7 indicate no clinically significant insomnia, 8–14 reflect subthreshold insomnia, 15–21 indicate moderate insomnia, and 22–28 represent severe insomnia.

Secondary outcomes encompassed psychological, quality-of-life, gastrointestinal, and overall well-being measures. The Generalized Anxiety Disorder 7-item (GAD-7, [39]) and the Patient Health Questionnaire 9-item (PHQ-9, [40,41]) were used to assess psychological symptoms. A higher score indicates worse mental health status. The quality of life was evaluated using the Short Form of Quality of Life, Enjoyment, and Satisfaction Questionnaire (QLESQ-SF, [42,43]), with a total score ranging from 14 to 70. Higher scores indicate more satisfaction with life. We also used the Visual Analog Scale of Gastrointestinal Discomfort (VAS-GI, [44,45,46]) to assess participants’ gastrointestinal symptoms. Respondents rated the severity of symptoms, including dry mouth, difficulty swallowing, loss of appetite, nausea and vomiting, bloating, stomachache, upper and lower abdominal pain, constipation, and diarrhea, on a 10 cm visual analog scale. Each symptom was scored from 0 to 10, yielding a total possible score of 0 to 100, with higher scores indicating greater symptom severity. Moreover, the Patient Global Impression of Change (PGI-C, [47]) was also utilized to evaluate participants’ subjective perception of overall change following the intervention. Respondents rated their perceived change on a 7-point Likert scale, ranging from “very much improved”, “much improved”, and “minimally improved”, to “no change”, “minimally worse”, “much worse”, or “very much worse.”

### 2.4. Subjective and Objective Sleep Assessments

We utilized commercial wrist actigraphy, Fitbit Inspire 3 (Firmware version 20001.188.58; Google Fitbit, San Francisco, CA, USA), and a sleep log to assess participants’ circadian rhythm. A Fitbit Inspire 3 was worn on the non-dominant wrist of each participant throughout the study period, except for showering and charging. The accuracy and reliability of Fitbit devices have been demonstrated in previous studies [48,49,50]. Participants were instructed to sync data through Fitbit app daily after waking. Sleep efficiency (SE), total sleep time (TST), time in bed (TIB), sleep onset latency (SOL), wake after sleep onset (WASO), sleep time of each sleep stage (light sleep, deep sleep, and rapid eye movement [REM] sleep), and a sleep quality score were extracted from Fitbit as the sleep parameters. Additionally, sleep hygiene includes time of sleep onset, wakeup time, nightmares, enough sleep, and snooze were recorded daily in a sleep log. An average score was calculated when data were available for three or more consecutive days per week.

### 2.5. Biomarkers

To minimize circadian variation, all biological samples were collected at consistent time points during both baseline (V1) and evaluation (V2) phases. Blood samples were collected in the morning (09:00–12:00), while saliva and urine samples were collected at night (21:00–23:00). The study targeted sleep-related biomarkers, including serum and salivary melatonin, cortisol, GABA, orexin, serotonin, as well as serum and urinary norepinephrine and urinary 6-sulfatoxymelatonin. Melatonin levels in serum were measured using the Human MT (Melatonin) ELISA Kit (MyBiosource, Inc., San Diego, CA, USA), and salivary melatonin was measured using the Melatonin direct Saliva ELISA Kit (IBL International GmbH, Hamburg, Germany). Cortisol in serum and saliva was quantified using the Elecsys Cortisol electrochemiluminescence immunoassay kit (Roche Diagnostics, Mannheim, Germany). Blood GABA levels were quantified with the Human GABA (Gamma-aminobutyric acid) ELISA Kit (Wuhan Fine Biotech Co., Ltd., Wuhan, China). Orexin levels were assessed with the Human Orexin ELISA Kit (MyBiosource, Inc., San Diego, CA, USA). Serotonin levels were assessed using the serotonin high-sensitive ELISA (LDN GmbH, Nordhorn, Germany). Norepinephrine was assessed using the NA/NE (Noradrenaline/Norepinephrine) ELISA Kit (Elabscience Biotechnology Inc., Houston, TX, USA). Urine 6-sulfatoxymelatonin was measured using the Melatonin-Sulfate Urine ELISA Kit (IBL International GmbH, Hamburg, Germany). All measurements followed the manufacturers’ instructions. Detailed assay characteristics, including sensitivity and intra-assay coefficients of variation, are provided in Supplementary Table S1.

### 2.6. Statistical Analysis

A compliance rate of 80% or higher was set for the per-protocol analysis. Categorial and continuous variables were analyzed using Pearson’s chi-squared tests and independent *t*-tests separately to compare the baseline characteristics and baseline performance. We utilized generalized estimating equations (GEE) models to investigate the effectiveness of treatment, with the Bonferroni method as post hoc correction. The unstandardized beta coefficient, 95% CI, and *p* value were reported in the results. We also exploratorily analyzed the potentiality of biomarkers through calculating the down/upregulation proportion by Pearson’s chi-squared tests. We used SPSS 20.0 to perform all the analyses and set the significance level at α = 0.05.

## 3. Results

### 3.1. Demographic Characteristics and Baseline Performances

**Figure 1:**
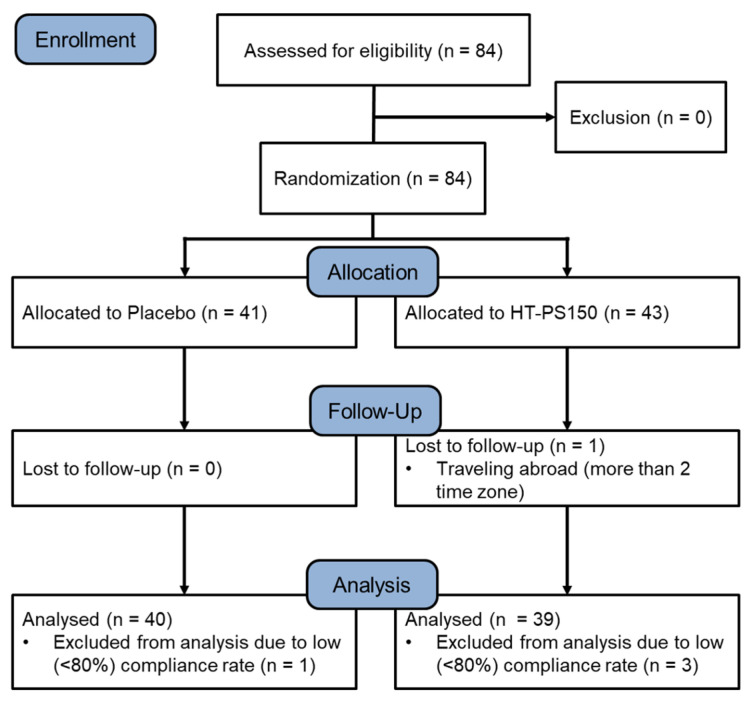
The flow diagram of the study. A total of 84 participants were enrolled and randomly distributed to the placebo group (*n* = 41) and the HT-PS150 group (*n* = 43). Per protocol analyses were conducted after excluding those who dropped out or with low compliance rates (<80%).

Eighty-four eligible participants were enrolled and randomly assigned to the placebo group (*n* = 41) or the HT-PS150 group (*n* = 43). One participant in the HT-PS150 group was lost to follow-up due to long-distance travel. Moreover, one participant in the placebo group and three participants in the HT-PS150 group with low compliance rates were also excluded from the analyses (Figure 1). As shown in Table 1, both groups had comparable demographic characteristics (*p* > 0.05). The sleep quality and insomnia symptoms as revealed by PSQI global score and ISI total score at baseline showed no significant difference between groups (*p* = 0.837 and *p* = 0.372, respectively; Table 1). In addition, throughout the study, participants experienced no adverse events, indicating that the intervention was well tolerated.

### 3.2. Efficacy of HT-PS150

Table 2 shows that the primary outcomes, the PSQI global score and ISI total score, significantly decreased over time (*p* = 0.006 and *p* < 0.001, respectively). However, the time × group interaction of PSQI analyzed by GEE was not significant (*p* = 0.921 and *p* = 0.794, respectively). In addition, the remaining variables showed no significant interactions (Table 2 and Table S2, and Supplementary Figure S1), implying that there were no considerable differences in psychological disturbance, gastrointestinal symptoms, circadian evaluations or biomarkers after the 8-week treatment. Furthermore, the results of the PGI-C showed no significant difference between the placebo and HT-PS150 groups, with a *p*-value from the chi-squared test of 0.283 (Table S3).

### 3.3. Results of Biomarker Regulation

**Figure 2:**
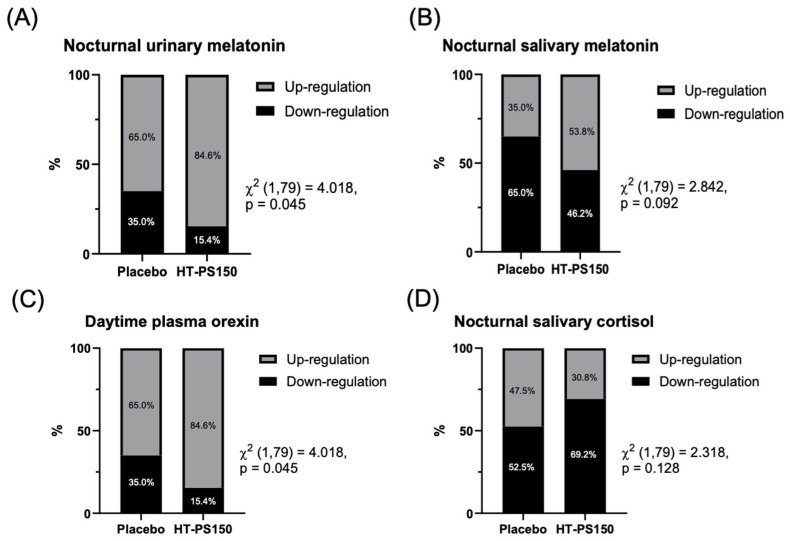
Up/down-regulation proportion comparison of biomarkers. Each participant’s regulations of biomarkers (Nocturnal urinary melatonin (**A**), salivary melatonin (**B**), daytime plasma orexin (**C**), and nocturnal salivary cortisol (**D**)) were calculated by subtracting posttest value (V2) from pretest value (V1). The number of participants showing down-regulation (value ≤ 0) and up-regulation (value > 0) was compared between groups with Pearson’s chi-squared test. Urinary melatonin values were corrected by creatinine clearance rate.

The tested biomarkers, including melatonin, cortisol, GABA, orexin, serotonin, norepinephrine and urinary melatonin, did not show any significant differences in absolute values between groups (Figure S1). However, as shown in Figure 2, 84.6% of participants in the HT-PS150 group, compared with 65.0% in the placebo group, exhibited an increase in nighttime urinary melatonin following treatment (*p* = 0.045). The result of nighttime salivary melatonin also showed a similar trend with a higher proportion of participants showing up-regulation in the HT-PS150 group (53.8%) than the placebo group (35.0%) (*p* = 0.092). Moreover, 84.6% of participants in the HT-PS150 group showed an improvement in daytime plasma orexin levels, compared to 65.0% in the placebo group (*p* = 0.045). Lastly, a higher proportion of participants in the HT-PS150 group (69.2%) revealed a reduction in nighttime salivary cortisol compared with the placebo group (52.5%) (*p* = 0.128).

### 3.4. Subgroup Results of Those with Higher Insomnia Symptoms (ISI ≥ 8)

**Figure 3:**
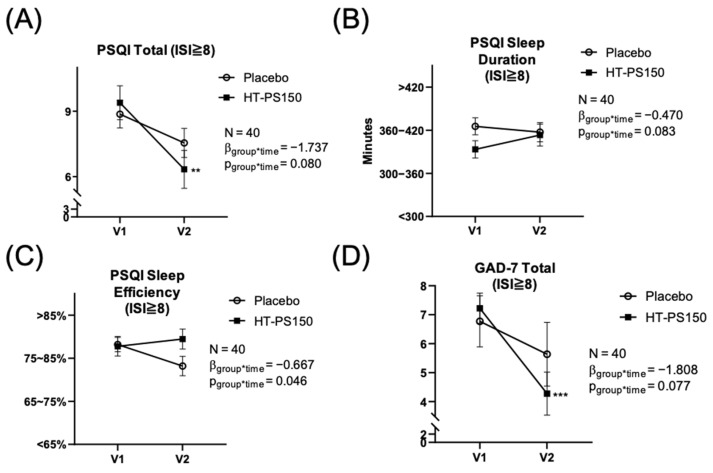
The results of subgroup analyses. (**A**) Total PSQI score, (**B**) PSQI sleep duration time, (**C**) PSQI sleep efficiency and (**D**) GAD-7 score. Subgroup analyses were conducted of those who scored 8 or higher on the ISI total score at screening (V0), which were *n* = 22 in the placebo and *n* = 18 in the HT-PS150. Unstandardized beta and *p* value were presented as the results of generalized estimating equations without any covariates. Post hoc (Bonferroni method) results were presented as within-group (versus HT-PS150 at V1), *p* ** < 0.01, *p* *** < 0.001. Abbreviation: PSQI = Pittsburgh Sleep Quality Index, ISI = Insomnia Severity Index, GAD-7 = Generalized Anxiety Disorder-7.

Individuals with more severe insomnia symptoms experienced greater sleep disturbances. Therefore, a subgroup analysis was conducted for participants with an ISI total score ≥ 8 at screening (V0), corresponding to subthreshold to moderate insomnia. As shown in Figure 3A, the GEE analysis revealed a trend toward a significant interaction effect for the PSQI total score (*p* = 0.080). Post hoc analysis revealed a significant reduction at V2 compared with V1 in the HT-PS150 group (*p* = 0.002). Furthermore, the sub-scores of PSQI indicated greater improvement in the HT-PS150 group compared with the placebo group, especially in sleep duration and sleep efficiency (*p* = 0.083 and 0.046, respectively; Figure 3B and Figure 3C). Lastly, as shown in Figure 3D, a trend toward improvement in GAD-7 was observed (*p* = 0.070). Within the HT-PS150 group, the total GAD-7 score was significantly reduced after treatment (V2) compared with baseline (V1), with a post hoc *p* < 0.001.

## 4. Discussion

Insomnia is mechanistically complex and tightly coupled to stress and mood systems [51]. Beyond nocturnal complaints, dysregulated sleep amplifies negative affect, heightens stress reactivity, and degrades cognitive control, which in turn worsens sleep and reinforces a cycle linking arousal systems, mood disturbance, and circadian misalignment [52]. In this randomized, double-blind, placebo-controlled trial of adults with poor sleep, eight weeks of HT-PS150 supplementation led to a higher proportion of participants exhibiting favorable endocrine responses related to sleep–wake regulation: increased nocturnal melatonin in urine and saliva, decreased nocturnal salivary cortisol, and improved daytime plasma orexin levels relative to placebo. By contrast, both groups showed improvements in sleep quality scores over time.

Interpreted as an integrated system, the alignment between self-perceived improvement and multi-matrix endocrine signals suggests biological engagement of the intervention at the individual level, even in the absence of group-level mean differences. Urinary melatonin reflects cumulative overnight pineal melatonin secretion and is considered a reliable surrogate of nocturnal circadian output [53]. Salivary melatonin provides a minimally invasive, time-specific measurement of the same circadian signal and is sensitive to changes in circadian phase and melatonin onset [54,55]. Nocturnal salivary cortisol indexes HPA-axis activation during the biological night and serves as a marker of hyperarousal [56]. Plasma orexin, while an imperfect proxy for central hypocretin tone, reflects daytime wakefulness drive and helps contextualize whether nighttime endocrine changes translate into altered daytime arousal [57]. Together, these matrices provide complementary insight into how an intervention may influence both nighttime sleep physiology and next-day arousal balance. Urinary melatonin integrates pineal output across the night, while salivary melatonin provides a minimally invasive readout of the same circadian signal; their concordant increase indicates enhancement of endogenous nocturnal melatonin secretion [58]. Concurrent lowering of nocturnal salivary cortisol points to attenuation of HPA-axis hyperarousal during the sleep period [59]. Daytime plasma orexin shifted as expected when nighttime melatonin was reinforced and evening cortisol was reduced, consistent with recalibration of the wake-promoting system, although peripheral orexin remains an imperfect surrogate for central hypocretin tone and should therefore be interpreted cautiously [60]. Collectively, these directions map onto canonical sleep biology, in which a stronger nocturnal melatonin signal couples with a quieter stress axis and a more appropriately tuned daytime arousal drive.

While the absolute biomarker changes were modest, the internal consistency across melatonin, cortisol, and orexin suggests physiological rather than stochastic variation. The concordant directionality across matrices (urine, saliva, plasma) strengthens interpretability and implies coordinated modulation of circadian and HPA-axis dynamics. Such multi-analyte coherence, despite limited mean separation, supports the view that HT-PS150 may engage regulatory feedback loops within the sleep–wake endocrine network rather than act on isolated targets. This physiological pattern is mechanistically plausible within the broader framework of the microbiota–gut–brain axis and consistent with the known properties of PS150. Gut microbes regulate the tryptophan–serotonin–melatonin pathway by shaping enterochromaffin-cell serotonin biosynthesis, producing short-chain fatty acids that modulate neuroinflammatory tone and influence sleep architecture, and communicate bidirectionally with the brain through vagal and autonomic routes [15,16]. Heat-treated postbiotics preparations preserve structural components such as lipoteichoic acids and peptidoglycans, which engage host pattern-recognition receptors and can rebalance immune signaling without microbial viability, providing a biologically plausible mechanism for lowering evening HPA axis drive [61]. Preclinical work with PS150 has demonstrated shorter sleep latency, increased non-rapid eye movement (NREM) and total sleep, mitigation of caffeine-induced insomnia, and faster rapid eye movement (REM) recovery, aligning with an anti-hyperarousal profile [32,33]. Experimental evidence further shows that melatonin inhibits orexinergic neurons via MT1 receptor-dependent mechanisms, offering a parsimonious link between strengthened nocturnal melatonin signaling to a tempered wake-promoting system [62]. In parallel, chronic sleep disturbance is known to upregulate pro-inflammatory cytokines and activate the HPA axis, creating a stress–inflammation–insomnia loop that microbiota-targeted modulation could attenuate [16,63].

The broader literature is consistent with this study. Meta-analytic syntheses generally report modest improvements in subjective sleep indices with probiotic or postbiotic interventions in people with insomnia or suboptimal sleep, with variable effects on objective measures and substantial heterogeneity, a pattern typical of strain-specific and phenotype-dependent responses [64,65]. Single-trial data reinforce the same nodes: *Lactiplantibacillus plantarum* JYLP-326 improved both sleep and mood in students, accompanied by microbial or metabolite shifts, and a randomized trial of prebiotic yeast mannan-oligosaccharide improved gut health and sleep quality in healthy adults, alongside changes in propionate and GABA [27]. Serotonergic pathways are biologically plausible in this context, although not always directly assayed [66]. Against this backdrop, melatonin-centric and HPA-axis readouts function as sensitive intermediates linking microbial modulation to sleep outcomes and are congruent with the endocrine pattern observed here.

Several limitations qualify the interpretation. The sample size was modest and drawn from a community cohort with generally mild baseline symptoms, a context that amplifies placebo and monitoring effects and produces floor effects in patient-reported outcomes and several biomarkers, thereby diluting between-group means even when individual trajectories move in biologically coherent directions. In addition, the proportional analyses of biomarker up- or down-regulation were exploratory and hypothesis-generating rather than confirmatory. The relatively small sample sizes available for biomarker analyses and subgroup comparisons further limit statistical power and increase susceptibility to chance findings, and therefore these results should be interpreted with caution. Although the per-protocol sample size was acceptable for an exploratory clinical trial, it may have limited statistical power to detect small between-group effects, particularly for secondary outcomes and biomarker analyses. Objective sleep was measured with a consumer wearable (Fitbit Inspire series) that performs reasonably well for total sleep time and sleep efficiency at the group level but has lower precision for sleep staging, wake after sleep onset, and latency, so small physiological gains may have been underestimated [50]. In addition, electroencephalography was not employed; therefore, the present study cannot determine whether HT-PS150 influences NREM sleep architecture, including slow-wave (stage 3) sleep. Future studies incorporating polysomnography are needed to directly evaluate this possibility. Peripheral orexin assays do not necessarily mirror central hypocretin dynamics and should be interpreted alongside melatonin and cortisol [67]. HT-PS150 is a heat-treated, non-viable preparation; therefore, its effects should not be assumed to mirror those of live PS150 or other probiotic formulations. The biological activity of postbiotics can depend on the specific strain, treatment process, and molecular composition, and thus the present findings should not be generalized to other preparations [68]. Methodologically, baseline PSQI/ISI scores and several biochemical indices clustered near physiological ranges, which limited headroom for mean change; similar psychobiotic and prebiotic trials have reported non-significant mean differences despite directional shifts consistent with biological activity. To preserve within-person signal under these constraints, we complemented conventional models with a proportional responder analysis that classified biomarker trajectories as up- or down-regulated and compared proportions between arms. This approach is less sensitive to non-normality and outliers and is informative when biochemical floors mute group-mean contrasts. The trends shown in Figure 2 and Supplementary Figure S1, which favor HT-PS150 across melatonin, cortisol, and orexin despite conservative means, align with this analytic context. Future studies should pre-register responder thresholds and multiplicity control, power on biomarker-defined response rates and circadian phase metrics, incorporate polysomnography with timed melatonin and cortisol sampling, and broaden eligibility to test generalizability.

In summary, this randomized, double-blind, placebo-controlled trial provides preliminary evidence that HT-PS150 may influence sleep-relevant endocrine/circadian pathways, potentially strengthening nocturnal melatonin signaling, as HT-PS150 was associated with a greater proportion of participants showing reductions in nocturnal salivary cortisol, suggesting attenuation of nighttime HPA-axis hyperarousal, and moderating wake drive, even though whole-cohort symptom means did not separate. Clinical improvements appeared more prominent among participants with greater baseline insomnia, suggesting possible responsiveness in those with mild to moderate symptom severity. These findings support the idea that HT-PS150 is safe and biologically active in pathways relevant to sleep regulation, and that it may hold potential as an adjunctive approach for individuals with mild to moderate insomnia. Larger, severity-enriched trials incorporating pre-specified responder criteria, polysomnography, and microbiome/metabolome profiling are warranted to confirm efficacy and elucidate mechanisms.

## 5. Conclusions

In adults with poor sleep, eight weeks of HT-PS150 supplementation did not differ from placebo in whole-cohort symptom means but was associated with a coherent endocrine signature across biological matrices, consistent with engagement of the sleep–wake regulatory system: increased nocturnal melatonin output, reduced nocturnal cortisol, and favorable adjustment of daytime orexin. Clinical benefits appeared more evident among participants with higher baseline insomnia, suggesting severity-dependent responsiveness and underscoring the strain-specific nature of postbiotic effects. Taken together, this exploratory randomized trial indicates that HT-PS150 did not demonstrate superiority over placebo in the overall study population. However, directionally favorable signals, particularly among participants with higher baseline insomnia severity, suggest potential biological engagement and warrant further investigation in larger, adequately powered trials.

## Figures and Tables

**Table 1:** Changes in Body Weight and Food Intake Among Experimental Groups During the Study Period.

Variables	Placebo	HT-PS150	*p*
	N (%) or M (SD)	N (%) or M (SD)	
Sex			0.926
Male	16 (40.0%)	16 (41.0%)	
Female	24 (60.0%)	23 (59.0%)	
Marital status			0.504
Single	25 (62.5%)	18 (46.2%)	
Cohabit	1 (2.5%)	1 (2.6%)	
Married	12 (30.0%)	17 (43.6%)	
Separated	1 (2.5%)	0 (0.0%)	
Divorced	1 (2.5%)	2 (5.1%)	
Widowed	0 (0.0%)	1 (2.6%)	
Children			0.314
With	16 (40.0%)	20 (51.2%)	
Without	24 (60.0%)	19 (48.8%)	
Other supplements			0.391
With	16 (40.0%)	12 (30.8%)	
Without	24 (60.0%)	27 (69.2%)	
Age	34.1 (11.4)	36.3 (11.1)	0.380
Education	16.2 (1.8)	16.5 (1.7)	0.466
BMI	23.9 (3.7)	23.9 (4.1)	0.973
PSQI Global	7.6 (2.2)	7.7 (2.8)	0.837
ISI Total	8.4 (4.0)	7.6 (3.8)	0.372

Continuous variables were analyzed by independent *t* test, and categorical variables were analyzed by Pearson’s chi-squared test. Abbreviation: BMI = body mass index, PSQI = Pittsburgh Sleep Quality Index, ISI = Insomnia Severity Index.

**Table 2:** GEE results of psychological inventories (N = 79).

Variables	V1		V2		Group		Time		Group*Time	
	Placebo	HT-PS150	Placebo	HT-PS150						
	M (SD)	M (SD)	M (SD)	M (SD)	β [95% CI]	*p*	β [95% CI]	*p*	β [95% CI]	*p*
PSQI Global	7.7 (3.2)	7.6 (3.1)	6.5 (2.8)	6.4 (2.8)	−0.110 [−1.490, 1.269]	0.876	−1.250 [−2.147, −0.353]	0.006	0.071 [−1.317, 1.458]	0.921
ISI Total	9.3 (4.7)	8.9 (4.1)	6.4 (4.5)	6.4 (4.7)	−0.326 [−2.251, 1.599]	0.740	−2.875 [−4.409, −1.341]	<0.001	0.285 [−1.855, 2.425]	0.794
GAD-7 Total	5.1 (4.1)	5.5 (3.1)	4.4 (4.3)	3.6 (2.8)	0.412 [−1.172, 1.995]	0.610	−0.625 [−1.616, 0.366]	0.217	−1.196 [−2.563, 0.172]	0.087
PHQ-9 Total	5.5 (4.1)	5.6 (3.4)	4.0 (4.3)	4.2 (2.9)	0.141 [−1.500, 1.782]	0.866	−1.475 [−2.632, −0.318]	0.012	0.013 [−1.555, 1.582]	0.987
QLESQ-SF Total	51.0 (8.1)	48.8 (6.2)	52.4 (8.4)	50.2 (5.7)	−2.154 [5.288, 0.979]	0.178	1.450 [−0.592, 3.492]	0.164	−0.117 [−3.281, 3.048]	0.942
VAS-GI Total	11.1 (9.7)	12.7 (10.4)	9.1 (9.5)	12.1 (11.1)	1.669 [−2.706, 6.044]	0.455	−2.025 [−4.787, 0.737]	0.151	1.333 [−2.917, 5.582]	0.539

Unstandardized beta and *p* value were presented as the results of generalized estimating equations (GEE) without any covariable. The dummy variables were set as group (0 = placebo, 1 = HT-PS150) and time (0 = V1, 1 = V2). Abbreviation: PSQI = Pittsburgh Sleep Quality Index, ISI = Insomnia Severity Index, GAD-7 = Generalized Anxiety Disorder-7, PHQ-9 = Patient’s Health Questionnaire-9, QLESQ-SF = Quality of Life Enjoyment and Satisfaction Questionnaire-Short Form.

## Data Availability

The original contributions presented in this study are included in the article/Supplementary Materials. Further inquiries can be directed to the corresponding author.

## Supplementary Materials

The following supporting information can be downloaded at: https://www.mdpi.com/article/10.3390/nu18010014/s1, Figure S1: The results of biomarkers; Table S1: Characteristics of the immunoassay kits used in this study; Table S2: GEE results of actigraphy and sleep diary (N = 79); Table S3: Results of PGI-C at V2.
