# Supplementary material for: Heat-Treated Limosilactobacillus fermentum PS150 Improves Sleep Quality with Severity-Dependent Benefits: A Randomized, Placebo-Controlled Trial
Source: Nutrients. 2025 Dec 19;18(1):14. doi: 10.3390/nu18010014 (PMC12787598; doi:10.3390/nu18010014)
Supplement: Supplementary file 1 [file nutrients-18-00014-s001.zip › Supplementary Figure 1.pdf]

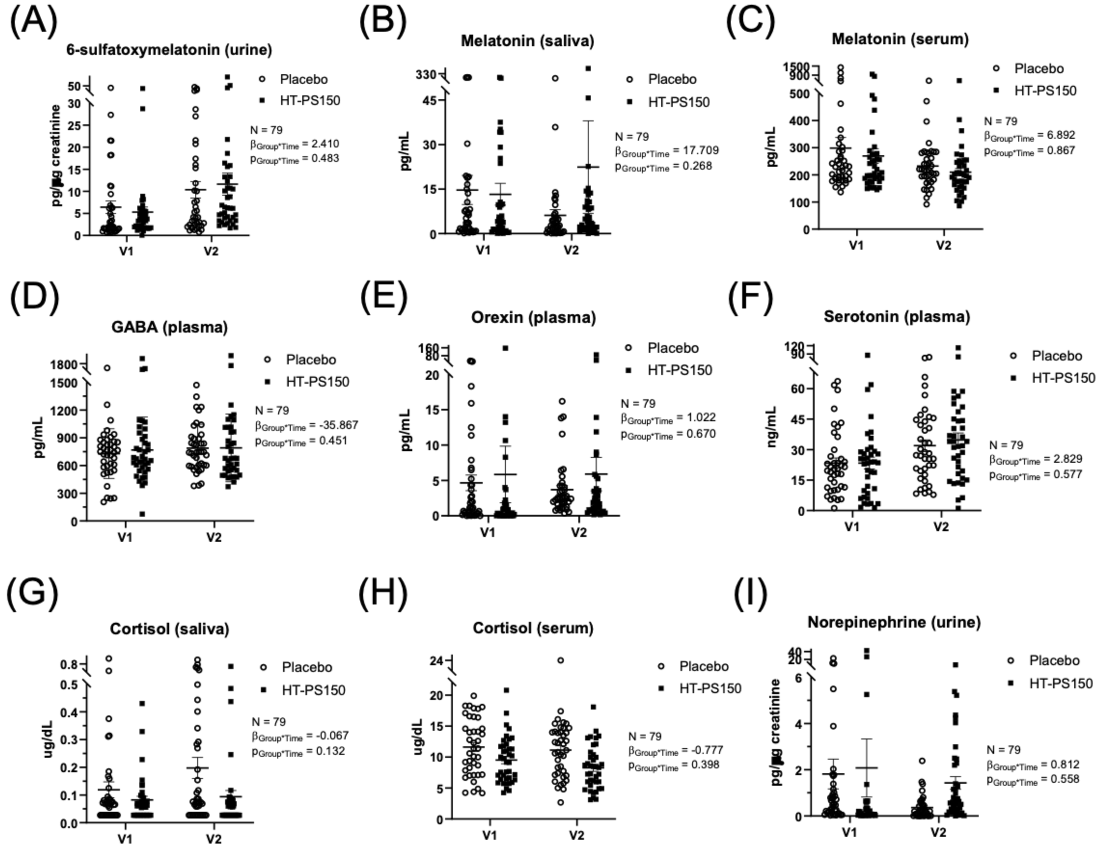

Supplementary Figure 1. The results of biomarkers. Unstandardized beta and p value were presented as the results of generalized estimating equations without any covariable. The dummy variables were set as group (0 = placebo, 1 = HT-PS150) and time (0 = V1, 1 = V2). Unary and salivary samples were collected at nighttime (21:00-23:00. Blood samples were collected at daytime (09:00-12:00). (1A & 1I) The value of Unary 6-sulfatoxymelatonin and Norepinephrine were corrected by creatine clearance rate.
