# Supplementary material for: Heat-Treated Limosilactobacillus fermentum PS150 Improves Sleep Quality with Severity-Dependent Benefits: A Randomized, Placebo-Controlled Trial
Source: Nutrients. 2025 Dec 19;18(1):14. doi: 10.3390/nu18010014 (PMC12787598; doi:10.3390/nu18010014)
Supplement: Supplementary file 1 [file nutrients-18-00014-s001.zip › Supplementary Table 1.pdf]

Supplementary Table 1. Characteristics of the immunoassay kits used in this study

Reagent / Kit	Sensitivity	Assay coefficients	Catalog	Manufacturer
Human MT (Melatonin)	4.688 pg/ml	Low: 5.2%<br>Medium: 4.84%<br>High: 5.1%	MBS766108	MyBiosource, Inc, San Diego, CA, USA
Melationin direct Saliva	0.85 pg/mL	13.2% – 19.0%	RE54041	IBL International GmbH, Hamburg, Germany
Cortisol (serum and saliva)	0.054 μg/dL	Serum: < 2.6% and < 5.8%<br>Saliva: < 4.4% and < 10.9%	Elecsys Cortisol II	Roche e801, Roche Diagnostics, Mannheim, Germany
Human GABA (Gamma-aminobutyric acid)	18.75 pg/mL	Intra< 8%<br>Inter< 10%	EH3098	Fine Biotech Co., Ltd, Hubei, China
Human Orexin (OX)	5.04pg/mL	Intra<10%<br>Inter<12%	MBS2000168	MyBiosource, Inc, San Diego, CA, USA
SEROTONIN high sensitive	5 pg/ml	Intra and inter < 10%	BA E-5900R	LDN GmbH, Nordhorn, Germany
Noradrenaline/Norepinephrine	0.19 ng/mL	Intra and inter < 10%	E-EL-0047	Elabscience Biotechnology Inc, Houston, TX, USA
Melatonin-Sulfate Urine	0.19 ng/mL	Intra and inter < 10%	RE54031	IBL International GmbH, Hamburg, Germany
