# Supplementary material for: Heat-Treated Limosilactobacillus fermentum PS150 Improves Sleep Quality with Severity-Dependent Benefits: A Randomized, Placebo-Controlled Trial
Source: Nutrients. 2025 Dec 19;18(1):14. doi: 10.3390/nu18010014 (PMC12787598; doi:10.3390/nu18010014)
Supplement: Supplementary file 1 [file nutrients-18-00014-s001.zip › Supplementary Table 2.pdf]

Supplementary Table 2. GEE results of actigraphy and sleep diary (N = 79)

Variables	V1		V2		Group		Time		Group*Time	
	Placebo	HT-PS150	Placebo	HT-PS150						
	M (SD)	M (SD)	M (SD)	M (SD)	β [95% CI]	p	β [95% CI]	p	β [95% CI]	p
Actigraphy SE[1] (%)	86.8 (2.0)	86.8 (2.3)	87.1 (2.5)	86.4 (2.2)	0.000 [-0.010, 0.009]	0.951	0.002 [-0.006, 0.011]	0.598	-0.007 [-0.018, 0.004]	0.230
Actigraphy TST[1] (mins)	375.7 (53.1)	355.4 (57.5)	382.3 (43.9)	351.6 (64.1)	-21.306 [-45.854, 3.243]	0.089	6.597 [-10.274, 23.469]	0.443	-9.356 [-36.279, 17.566]	0.496
Actigraphy TIB[1] (mins)	432.8 (62.7)	408.9 (68.6)	439.6 (51.3)	407.5 (74.0)	-23.881 [-53.015, 5.252]	0.108	6.768 [-12.271, 25.807]	0.486	-8.198 [-39.670, 23.273]	0.610
Actigraphy WASO[1] (mins)	57.6 (13.3)	55.4 (15.9)	58.2 (13.9)	56.5 (14.9)	-2.219 [-8.713, 4.275]	0.503	0.581 [-3.776, 4.938]	0.794	0.493 [-6.529, 7.516]	0.890
Actigraphy SOL[1] (mins)	0.0 (0.2)	0.0 (0.0)	0.0 (0.0)	0.0 (0.0)	-0.031 [-0.091, 0.029]	0.311	-0.031 [-0.091, 0.029]	0.311	0.031 [-0.029, 0.091]	0.311
Actigraphy Deep Sleep[1] (%)	16.9 (5.2)	17.6 (4.0)	16.1 (5.0)	16.6 (4.8)	0.007 [-0.014, 0.027]	0.509	-0.008 [-0.023, 0.007]	0.289	-0.001 [-0.021, 0.019]	0.891
Actigraphy Light Sleep[1] (%)	56.0 (12.2)	57.6 (11.2)	56.6 (12.7)	59.6 (12.1)	0.016 [-0.036, 0.068]	0.543	0.006 [-0.047, 0.059]	0.827	0.014 [-0.052, 0.080]	0.675
Actigraphy REM[1] (%)	20.0 (6.1)	20.0 (5.9)	19.8 (5.6)	18.2 (5.2)	0.000 [-0.027, 0.026]	0.997	-0.002 [-0.019, 0.016]	0.856	-0.016 [-0.041, 0.008]	0.196
Actigraphy Sleep Score[1]	64.0 (17.2)	66.6 (14.0)	71.2 (11.9)	68.8 (10.7)	2.630 [-4.355, 9.615]	0.461	7.241 [1.255, 13.226]	0.018	-5.071 [-12.727, 2.585]	0.194
Actigraphy Restlessness[1]	0.1 (0.0)	0.1 (0.0)	0.1 (0.0)	0.1 (0.0)	0.008 [-0.006, 0.021]	0.261	0.005 [-0.005, 0.015]	0.309	-0.002 [-0.015, 0.011]	0.735
Diary TIB	463.2 (63.4)	412.3 (49.5)	472.5 (70.5)	407.3 (50.7)	-54.224 [-80.033, -28.416]	<0.001	7.239 [-11.307, 25.784]	0.444	-23.722 [-58.147, 10.704]	0.177
Diary Nightmare	19.6 (33.4)	8.8 (22.6)	18.4 (34.3)	10.9 (24.4)	-0.094 [-0.220, 0.032]	0.144	-0.023 [-0.084, 0.038]	0.457	0.044 [-0.053, 0.140]	0.375
Diary Enough Sleep	41.1 (36.2)	28.6 (32.3)	47.6 (35.6)	32.3 (37.5)	-0.122 [-0.278, 0.035]	0.129	0.066 [-0.022, 0.154]	0.140	-0.034 [-0.163, 0.096]	0.612
Diary Snooze	53.9 (40.2)	51.4 (35.6)	43.8 (40.3)	45.2 (38.6)	-0.033 [-0.203, 0.138]	0.709	-0.118 [-0.228, -0.007]	0.037	0.031 [-0.111, 0.173]	0.668
Diary Overall	71.6 (8.6)	70.3 (8.4)	75.1 (8.1)	70.4 (9.1)	-1.892 [-5.843, 2.059]	0.348	2.985 [0.928, 5.043]	0.004	-2.750 [-6.231, 0.731]	0.122

Unstandardized beta and p value were presented as the results of generalized estimating equations (GEE) without any covariable. The dummy variables were set as group (0 = placebo, 1 = HT-PS150) and time (0 = V1, 1 = V2). [1]Three participants in the placebo group were excluded due to the lack of actigraphy data (less than 3 days per week). Abbreviation: SE = Sleep efficiency, TST = Total sleep time, TIB = Time in bed, WASO = Wake after sleep onset, SOL = Sleep onset latency, REM = Rapid eye movement
