# Supplementary material for: Heat-Treated Limosilactobacillus fermentum PS150 Improves Sleep Quality with Severity-Dependent Benefits: A Randomized, Placebo-Controlled Trial
Source: Nutrients. 2025 Dec 19;18(1):14. doi: 10.3390/nu18010014 (PMC12787598; doi:10.3390/nu18010014)
Supplement: Supplementary file 1 [file nutrients-18-00014-s001.zip › Supplementary Table 3.pdf]

Supplementary Table 3. Results of PGI-C at V2

PGI-C	Placebo	HT-PS150	*p*
	N (%)	N (%)	
Very Much Improved	0 (0.0%)	2 (5.1%)	
Much Improved	6 (15.0%)	2 (5.1%)	
Minimally Improved	18 (45.0%)	17 (43.6%)	0.283
No Change	16 (40.0%)	17 (43.6%)	
Minimally Worse	0 (0.0%)	1 (2.6%)	

Pearson's chi squared test was utilized to compare the difference between groups. Abbreviation: PGI-C = Patient's Global Impression of Change
